# Pollution Gradients Altered the Bacterial Community Composition and Stochastic Process of Rural Polluted Ponds

**DOI:** 10.3390/microorganisms8020311

**Published:** 2020-02-24

**Authors:** Xin Tai, Rui Li, Bao Zhang, Hao Yu, Xiao Kong, Zhihui Bai, Ye Deng, Lan Jia, Decai Jin

**Affiliations:** 1 College of Environmental Science and Engineering, Liaoning Technical University, Fuxin 123000, China; taixin_xin@163.com; 2 CAS Key Laboratory of Environmental Biotechnology, Research Center for Eco-Environmental Sciences, Chinese Academy of Sciences, Beijing 100085, China; ruili_st@rcees.ac.cn (R.L.); Mrzhangbao08@163.com (B.Z.); zhbai@rcees.ac.cn (Z.B.); yedeng@rcees.ac.cn (Y.D.); 3 College of Resources and Environment, University of Chinese Academy of Sciences, Beijing 100049, China; 4 School of Civil Engineering, Lanzhou University of Technology, Lanzhou 730050, China; 5 School of Health and Public, Qingdao University, Qingdao 266071, China; kongxiaozaikeda@163.com; 6 Research Institute of Mineral Resources Development and Utilization Technology and Equipment, Liaoning Technical University, Fuxin 123000, China; jialan.lan@163.com

**Keywords:** rural polluted ponds, livestock wastewater, high-throughput sequencing, bacterial community, community assembly mechanism

## Abstract

Understanding the effects of pollution on ecological communities and the underlying mechanisms that drive them will helpful for selecting a method to mediate polluted ecosystems. Quantifying the relative importance of deterministic and stochastic processes is a very important issue in ecology. However, little is known about their effects on the succession of microbial communities in different pollution levels rural ponds. Also, the processes that govern bacterial communities in polluted ponds are poorly understood. In this study, the microbial communities in water and sediment from the ponds were investigated by using the 16S rRNA gene high-throughput sequencing technology. Meanwhile, we used null model analyses based on a taxonomic and phylogenetic metrics approach to test the microbial community assembly processes. Pollution levels were found to significantly alter the community composition and diversity of bacteria. In the sediment samples, the bacterial diversity indices decreased with increasing pollutant levels. Between-community analysis revealed that community assembly processes among water and sediment samples stochastic ratio both gradually decreased with the increased pollution levels, indicating a potential deterministic environmental filtering that is elicited by pollution. Our results identified assemblage drivers of bacterial community is important for improving the efficacies of ecological evaluation and remediation for contaminated freshwater systems.

## 1. Introduction

Freshwater ecosystems play a key role in the efflux of carbon dioxide (CO_2_), methane (CH_4_), and the storage of organic carbon in sediments [1,2]. Ponds are defined as lentic water bodies < 2 ha in the United Kingdom and most of Europe [3]. There are approximately 4.2 million km^2^ of natural lakes and ponds on the surface of the earth, and the farm ponds cover about 77,000 km^2^ [2]. Ponds are among the most biodiverse and ecologically important freshwater habitats, which could provide habitats to endangered wetland plants, invertebrates, and amphibians. In addition, ponds provide flood alleviation, rainfall interception, and a supply of irrigation water [4,5,6]. However, with the development of livestock breeding, livestock wastewater seriously contaminated a large number of rural ponds and threatened human health and ecosystem security, which was considered to be the third largest water pollution source after industrial and domestic pollution [7]. Livestock wastewater contains a great number of pathogens which could cause various diseases, such as diarrheal diseases, heart attacks, insulin dependent diabetes (Coxsackie B virus), Guillain-Barre syndrome (paralysis, Campylobacter), and hemolytic uremic syndrome (HUS, *Escherichia coli* O157:H7) [8]. Especially in rural areas, most livestock wastewater is discharged directly into ponds without treatment, which could cause epidemics and is harmful to human health. 

Microbial communities play an essential role in the cycling of nutrients in all ecosystems. For example, some autotrophic bacteria taxa are thought to contribute towards carbon cycling [7]. As an important part of bioremediation, microbial agents can be used alone, or in combination with plants or other technologies, which play an important role in the degradation of water pollutants. The structure and composition of microbial community are related to water pollution status and are widely used as bioindicators of pollution levels. For instances, Li et al. [9] has selected 13 families and 9 orders as crucial indicator groups for different levels of eutrophication in Taihu Lake. Many previous studies also revealed that in freshwater systems, microbial biomass, activities, and structures shift spatially with water or sediment characteristics, such as pH, TN, heavy metals, etc. [10,11,12,13,14,15,16,17]. Yin et al. [18] indicated the phylogenetic diversity and structure of microbial communities would shift under heavy metal contamination to increase their adaptability and/or resistance to environmental contamination. Bier et al. [19] demonstrated the composition and diversity of microbial communities changed along chemical gradients in Central Appalachian streams, which indicated that microbial community information can be used to identify new gradient features. Overall, in a dynamic and complex freshwater ecosystem, the microbial community is a sensitive indicator for assessing the health of an ecosystem. According to some key microorganisms, it can track the changes in the degree of pollution in the aquatic ecosystem, thus providing a new type of monitoring for artificially affected freshwater systems. However, there is no information on the microbial community structure changes to the pollution which has increased in rural ponds.

Long-term contamination not only altered the biodiversity and spatial pattern of microorganisms, but also changed the mechanism of biological assembly [20]. Recently, information about the process and factors controlling community assembly has been essential to understanding the patterns of species composition and diversity. Two types of processes: niche-based (deterministic) and neutral (stochastic) processes, were often used to assay bacterial community assembly [21]. Stochastic processes include random changes in species’ relative abundances (ecological drift), colonization, extinction, and speciation [22,23]; deterministic processes include niche differentiation, ecological selection, and interspecific interactions, imposed by abiotic and biotic factors which influence organism fitness and thereby determine the composition and relative abundance of species [24,25,26]. At present, many researchers generally believed that both deterministic processes and stochastic processes played important roles during bacterial assembly [27,28]. It is also known that productivity, disturbance, and resource availability influence the relative importance of stochastic vs. deterministic processes in the assembly of local communities [29,30]. For example, a long-term experiment in replicate ponds showed that higher β-diversity at higher productivity resulted from a stronger role for stochastic relatives [31]. Stochastic factors are relatively more important when the local environmental conditions can become more benign. Meanwhile, nutrient addition would enhance stochastic processes in shaping microbial community structures [32]. To some extent, understanding the stochastic and deterministic of microbial communities is helpful to understand or predict the trend of water pollution. Any ecosystem restoration programs must consider approaches to facilitate both processes to mediate ecological succession and achieve a desired state [32]. The relative importance of processes is dependent on species abundance [33]. At a tall-grass prairie ecosystem, the relative importance of stochastic processes in governing bacteria community structure has decreased substantially over time under climate warming [34]. However, few studies focus on the variation of bacterial community assembly in the pollution gradients of rural ponds influenced by livestock wastewater; Whether the declining importance of stochastic processes are applicable to increasing pollution levels in freshwater ecosystems is not known. In this study, the response of the water and sediment microbial community to different levels of pollution in rural ponds, which is influenced by livestock wastewater, was investigated, and the assembly processes were also studied. We hypothesized that: (i) the structure, composition, and diversity of bacterial communities were greatly different between different pollutant levels. (ii) the relative importance of stochastic processes decreased over pollution levels, which were influenced by potential deterministic environmental filtering. To test the above hypotheses, microbial communities in water and sediment from different levels of polluted ponds were investigated and analyzed.

## 2. Materials and Methods

### 2.1. Sample Sites and Water Quality

The experimental sites were located at Xiongan New Area (Anxin County), Hebei province, China (38°10′–40°00′N, 113°40′–116°20′E), with a warm temperate semi-humid continental monsoon climate and four distinct seasons. The annual average temperature of these sites was 12.2 °C, with a maximum temperature of 40.7 °C in summer and a minimum temperature of −26.7 °C in winter. The average frost-free period was 203 days, and the annual average sunshine was 2578.3 hours, with an annual average precipitation of 529.7 mm. These ponds were polluted to different degrees by livestock wastewater. The wastewater was discharged from nearby pig farms, a small contributor to domestic wastewater and rainwater. We stopped wastewater from being discharged before sampling and it will not be discharged in the future. Detailed information for these ponds is shown in Table 1, while the locations of experimental sites are shown in Figure S1.

### 2.2. Sampling and Physical-Chemical Analysis

Three replicates of water and sediment samples were collected from eight ponds (within 2 m from the edge of the water body) in June 2018. These three sites had different distance from a sewage outlet. Water samples were collected from within 2 m from the edge of the water body at a depth of 30 cm and the sediment samples were collected at a depth of 15 cm below the sediment surface, as described previously [35,36,37]. These samples were collected in sterile containers and stored with ice bags immediately transported to the laboratory and stored in the dark at 4 °C until they were processed within 24 h. The pH of water samples was examined using potentiometry with a pH meter (PB-10, Sartorious, German). The content of total phosphorus (TP), total nitrogen (TN), and ammoniacal nitrogen (NH_3_-N) were measured by using UV-Vis spectrophotometry. The concentration of Chemical Oxygen Demand (COD) were measured by a spectrophotometer (DRB 200, Hach, USA). The sediment physiochemical properties were detected by the Pony Testing International Group (Beijing, China). The potential of hydrogen (pH) of the sediment was measured with a pH meter (PB-10, Sartorius, Germany). Sediment samples were dried with a laboratory freeze dryer (Scientz-10N, Ningbo Xinzhi Biological Technology Co., Ltd., Zhejiang, China). The concentrations of TP were measured using inductively coupled plasma mass spectrometry (ELAN 9000/DRC-e, PerkinElmer, Wellesley, Ma, USA). TN were measured with a CN analyzer (Vario Max CN; Elementar Analysensysteme, Hanau, Germany). The contents of Cu, Pb, Zn, Cd, Cr, and Ni were determined with an inductively coupled plasma mass spectrometer (ELAN9000, PerkinElmer SCIEX, Shelton, CT, USA). The concentrations of Hg and As was determined by using atomic fluorescence spectrometry (PF5 Atomic fluorescence photometer, Beijing Puxi General Instrument Co., Ltd., China). We classified the ponds into three groups by using the comprehensive pollution index (CPI) method based on the environmental variations of the water. It is an important method for water quality environmental assessment, which can comprehensively evaluate the water pollution status. The comprehensive pollution index was calculated by [38]:

(1) $$P=\frac{1}{n}\sum_{i=1}^{n}p_{i}$$

(2) $$p_{i}=C_{i}/S_{i}$$

where P is comprehensive pollution index, Pi is pollution index of the i-th pollutant, n is the types of pollutant, Ci is the average measured concentration of the i-th pollutant (mg/L or number/L), and Si is the evaluation standard value of the i-th pollutant (mg/L or number/L).

The results of the comprehensive pollution index in water samples are shown in Table 2. Based on the range of the CPI, the ponds were divided into three groups which are called mild, moderate, and severe polluted ponds, respectively.

### 2.3. DNA Extraction, PCR Amplification and High-Throughput Sequencing 

About 100 mL of water was pre-filtered through a 200 μm mesh to remove large metazoans and other particles, then filtered using a 0.22 μm pore-size polycarbonate membrane to collect the microorganism. To avoid minor stochastic mass variation, surface sludge was discarded before homogenization. The microbial genomic DNA of the water and sediment samples were extracted from 100 mL of water and 0.5 g of dry sediment using Fast DNA SPIN Kit for Soil (MP Biomedicals LLC, USA). Afterwards, the V4 region of the 16S rRNA gene was amplified using the primers 515F (5′-GTGCCAGCMGCCGCGGTAA-3′) and 806R (5′-GGACTACHVGGGTWTCTAAT-3′) for high-throughput sequencing, as described previously [39]. PCR amplification was performed using 50 μL of PCR mixture, including 5 μL of 10× PCR Buffer, 4 μL of dNTPs, 1 μL of forward and reverse primers (10 μM), 0.5 μL of Taq DNA polymerase (TaKaRa Biotech, Beijing, China), and 1 μL of template DNA. The final volume was adjusted to 50 μL with sterile water. PCR amplification was carried out with 5 min at 94 °C, followed by 30 cycles at 94 °C for 20 s, 57 °C for 25 s, primer extension at 68 °C for 10 min, and final by incubation for 10 min at 72 °C. The PCR products were purified by using E.Z.N.A.™ Gel Extraction Kit (Omega Bio-tek, Norcross, GA, USA). After purification, the PCR products were mixed with equimolar amounts prior to sequencing on an Illumina-Miseq platform developed by the Magigene Institute (Guangzhou, China).

Raw sequences were preprocessed and analyzed using an in-house Galaxy Pipeline (http://mem.rcees.ac.cn:8080/) [40], equipped with series of bioinformatics tools. FLASH [41] was used to combined forward and reverse sequences, and then sequences with length < 200 bp were deleted. UPARSE [42] was used to remove chimeras and to generate OTU (operational taxonomic unit) table at a 97% similarity level without any singletons being discarded. Afterwards the archaea sequences were removed from all sequences and the data were resampled randomly with the minimum sequences. In this study, 14,204 sequences for all samples were resampled as the minimum sequences. The resampled OTUs table was used for further statistical analyses.

The raw data of bacterial community have been submitted to NCBI sequence Read Archive (SRA) under the accession number SRP242481.

### 2.4. Statistical Analysis

The non-metric multidimensional scaling (NMDS) and dissimilarity tests based on analysis of similarity using the Bray Curtis distance were performed to evaluate the differences of microbial community structure between two groups. Canonical correspondence analysis (CCA) and mantel test were used to investigate the correlation between microbial community structures and environmental variables. All environmental variables were selected based on a variance inflation factor (VIF) < 20. A Venn Diagram was used to show the unique and shared OTUs detected in different groups. All the analyses were carried out in R project v.3.6.0 (www.R-project.org). The significant difference of environmental variables among three groups was calculated with one-way ANOVA analysis of variance by SPSS 21.

### 2.5. Community Assembly Processes in Three Groups of Ponds

To quantify the contributions of ecological stochasticity to microbial community structure under different pollutant levels ponds, we quantified the normalized stochasticity ratio (NST) using a pipeline (http://ieg3.rccc.ou.edu:8080/) [43]. NST is used to measures the relative position of observations under purely deterministic and purely stochastic. The value of NST > 50% indicates that stochasticity of community assembly was more important than the determinism, while NST < 50% means that more deterministic determinism of community assembly was more important than the stochasticity. The NST is calculated as

(3) NSSA=SSA−STSASDSA−STSA=∑ijnASSijA−mink{∑ijnAξ(Eij(k),Eij¯)}∑ijnA(1−Eij¯)−mink{∑ijnAξ(Eij(k),Eij¯)}

(4) NSSB=SSB−STSBSDSB−STSB=∑ijnBSSijB−mink{∑ijnBξ(Eij(k),Eij¯)}∑ijnBEij¯−mink{∑ijnBξ(Eij(k),Eij¯)}

(5) NSS =SS−STSSDS−STS=∑ijξ(Cij,Eij¯)−mink{ ∑ijξ(Eij(k),Eij¯)}∑ijξ(CDij,Eij¯)−mink{ ∑ijξ(Eij(k),Eij¯)}

(6) CDij={0   Cij<Eij¯1   Cij≥Eij¯ 

(7) $$\xi \left(\chi ,y\right)=\frac{\chi -y}{\chi -\delta }\;\delta ={\{}_{1\;\chi <y}^{0\;\chi \geq y\;}$$

(8) NST=1−NSS

where ^D^SS are completely deterministic and extreme values of SS, and ^T^SS is under stochastic assembly. The superscript A and B indicate type A and type B. DC_ij_ is the similarity under fully deterministic conditions about community i and j. It is also is one of the null expected values of similarity under fully stochastic assembly conditions about community i and j. ξ is a generalized function.

In our study, the estimated NST based on Jaccard (unweighted) similarity metrics and used the null model to estimate the magnitude of stochasticity in community assembly.

For further verification, the importance of stochastic processes underlying community assembly null model analysis was performed using abundance-based similarity metrics [32,34]. The significance of the difference around communities between the observed similarity matrices with the null model expectation was examined using a nonparametric permutation test, called the permutational multivariate analysis of variance (PERMANOVA) [23]. The calculations involved in stochastic ratio were performed by an in-house pipeline (http://mem.rcees.ac.cn:8080) [40]. In this procedure, an average null expected similarity and mean of observed similarity could be estimated based on 1000 drawings. If community assembly is primarily shaped by stochastic processes, then the similarity observed across the actual communities will be statistically indistinguishable from the random null expectation [23,32].

## 3. Results

### 3.1. Physiochemical Properties of Water and Sediment

According to the environmental quality of V standards for surface water issued by Chinese government in 2002 (GB3838-2002) [44], the concentration of NH_3_-N, COD, TN, and TP in water samples seriously exceeded the standard limit (Table 2). The comprehensive pollution index was calculated based on the physicochemical properties of water (*P* value). Using the national standard values, we took *P* ≥ 2.0 as the pollution index [38], then divided these ponds into three pollution gradients. When 0 < *P* ≤ 30, it was defined as mild pollution; moderate pollution was defined as 30 < *P* ≤ 60; *P* > 60 was defined as seriously polluted ponds. Concentrations of water pH, NH_3_-N, TP, COD, as well as TN showed three different trends in three group ponds. First, NH_3_-N, TP, and TN was significantly lower in the mild ponds compared with moderate and severe ponds, and was significantly lower in the moderate ponds compared with severe ponds. The values of TP and NH_3_-N were significantly different in the three groups of ponds. Second, pH does not exceed standards and mild ponds was significantly different between moderate and severe ponds. Third, COD was not significantly different between the three groups. Among the sediment samples (Table 3), there were no significant differences between the three groups of ponds. Compared with grade A of the Chinese Control Standards of Pollutants in sludge for Agricultural use (GB4284-2018) [45], we have found most heavy metals such as Cr, Ni, Cu, Zn, As, Cd, Pb, Hg, and OM (organic matter) met the limits required by national standards. In general, the water properties among different groups were different from each other, while sediment properties showed few differences.

### 3.2. Bacterial Community Structure of Three Group Ponds

**Figure 1:**
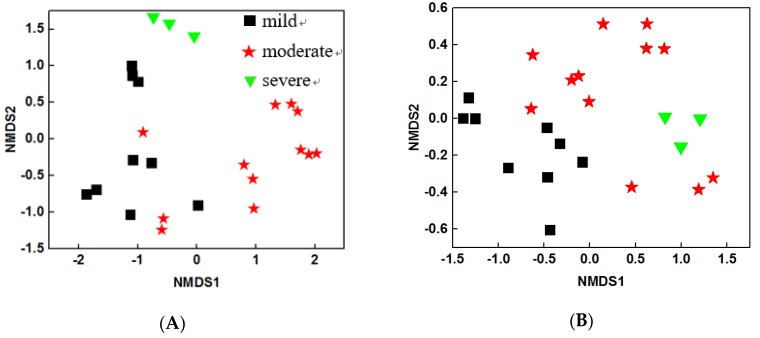
Non-metric multidimensional scaling plots based on the Bray Curtis index of (**A**) water samples and (**B**) sediment samples.

To characterize the structure of microbial community, NMDS analysis was performed based on Bray Curtis distance. The results showed that both water and sediment bacterial community structures were different among three pollutant levels (Figure 1A and Figure 1B). These results were further confirmed by two dissimilarity tests: multi-response permutation procedure (MRPP) and PERMANOVA based on Bray Curtis and Jaccard distances. The water bacterial communities were significantly different (*P* < 0.05) between any of the two pollutant levels (Table 4), suggesting that the pollutants may be the key factors that influence water bacterial community structures. Similar trends were observed for sediment bacterial communities, though the difference between moderate and severe samples were not significant (*P* = 0.092) during the PERMANOVA (Bray Curtis) analysis. Overall, the structures of water and sediment microbial communities were dramatically altered by the pollutant levels.

### 3.3. Diversity and Composition of Bacterial Community from Three Groups of Ponds

A total of 2,619,479 raw bacteria sequences were detected by using Illumina-MiSeq sequencing from 24 water and sediment samples. After a series of preprocessing, 2,131,351 qualified reads were classified into 33,342 OTUs at a 97% similarity level.

**Figure 2:**
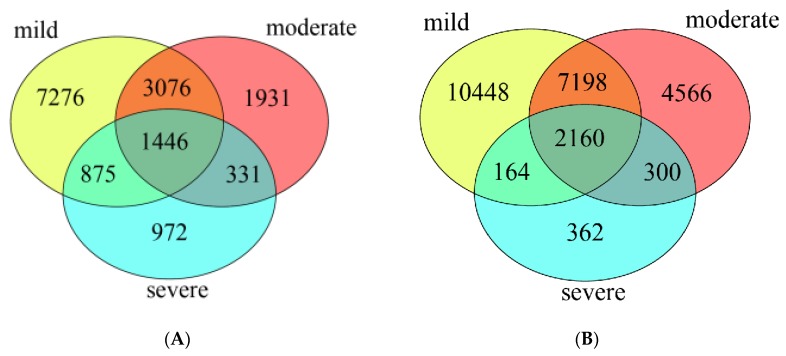
The unique and overlapped OTUs detected in water samples (**A**), and in sediment samples (**B**).

The Venn diagram showed that in water samples (Figure 2A), the bacterial community of three pollutant levels shared 1,446 OTUs, which accounted for 9.1% of the total OTUs. The mild polluted ponds had the highest number of unique OTUs (12,673, 79.7%), followed by moderate (6,784, 42.6%) and severe ponds (3,624, 22.8%). In sediment samples (Figure 2B), the bacterial community of three groups ponds shared 2,160 OTUs which accounted for 8.6% of the total OTUs. Similarly, the mild polluted ponds had the highest number of unique OTUs (19,970, 79.3%), followed by moderate (14,224, 56.4%), and the lowest for severe ponds (2,986, 11.9%).

**Figure 3:**
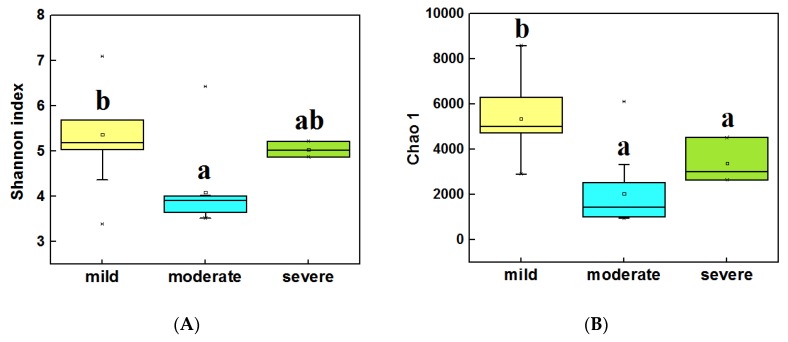

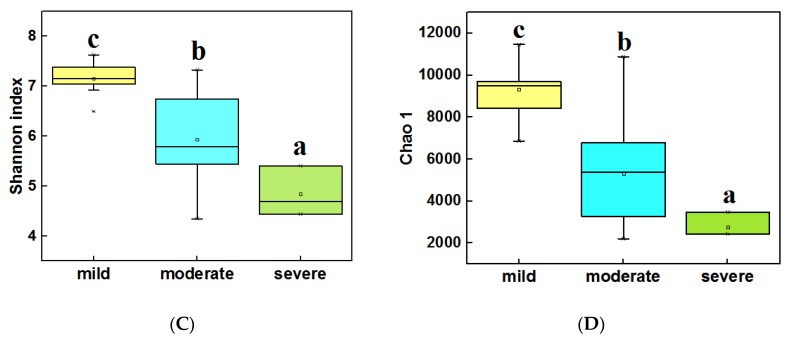
Comparisons of the 16S Shannon index in water samples of three group ponds (**A**). Comparison of 16S Chao1 value in water samples (**B**). Comparisons of 16S Shannon index in sediment samples (**C**). Comparison of 16S Chao1 value in sediment samples (**D**). Different letters are significantly different from one another under three groups, and the significance level is *P* < 0.05.

The alpha diversity (α-diversity) of bacterial communities was calculated by using the Shannon index and Chao 1. In water samples (Figure 3A,B), the highest α diversity was obtained from mild ponds, which was significantly higher than the other two groups (LSD test, *P* < 0.05). Meanwhile, the α diversity of severe ponds were higher than moderate ponds, though the moderate and severe ponds were not significantly different from each other. In sediment samples (Figure 3C,D), the highest α diversity were obtained from mild ponds. Meanwhile, the α diversity of moderate ponds was also significantly higher than for severe ponds, and there was significance of difference among three group ponds (LSD test, *P* < 0.05). These results indicated that the α-diversities of the bacterial communities were significantly decreased with the increasing pollutant level of the ponds.

In addition, the correlation analysis (Spearman correlation test) (Table S1) indicated that the Shannon index, Observed richness, and Chao 1 of bacterial communities were significantly correlated with pH (*P* < 0.05), meanwhile Observed richness and Chao 1 were also significantly correlated with TP and NH_3_-N (*P* < 0.05) in water samples. In sediment samples (Table S2) among the main environmental factors, only Cu and Zn were significantly correlated with the four diversity indexes.

**Figure 4:**
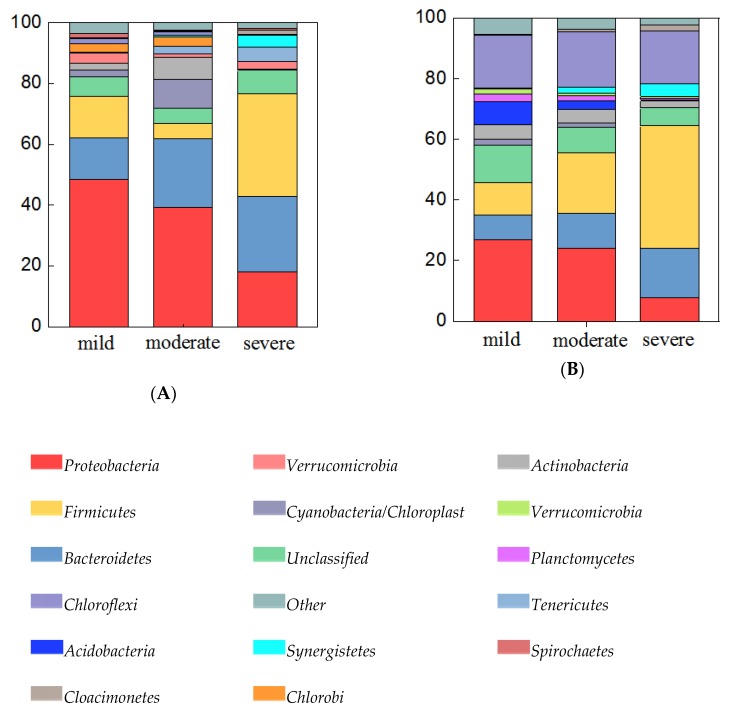
Bacteria abundance (abundance > 5%) at the phylum level in water samples (**A**), and sediment samples (**B**). Categories with abundance < 1% are summarized as “others”.

Both in water and sediment samples, the abundance of bacterial communities differed greatly at the phylum level among the three pollutant levels. In 24 water samples (Figure 4A), all of the detected 16S rRNA gene sequences belonged to 39 bacterial phyla. Among them, the predominant phyla (relative abundance > 5%) were *Proteobacteria* (18.0%–48.5%), *Bacteroidetes* (13.6%–25.0%), *Firmicutes* (5.1%–33.6%), *Cyanobacteria / Chloroplast* (0.1%–9.5%), and *Actinobacteria* (0.4%–7.2%). Among the three pollutant levels, the relative abundance of *Bacteroidetes* increased with increasing pollutant levels, while the abundance of *Proteobacteria* was decreased with increasing pollution levels. Specifically, the relative abundance of *Firmicutes* was highest in severely polluted ponds. The sediment bacterial community of samples (Figure 4B) included 41 phyla, and the *Firmicutes*, *Proteobacteria*, *Chloroflexi*, *Bacteroidetes*, *Thaumarchaeota*, *Cloacimonetes*, and *Synergistetes* were dominant phyla (relative abundance > 5%) which accounted for 78.94%–91.59% of the total OTUs. Relative abundances of *Firmicutes*, *Bacteroidetes*, *Cloacimonetes*, and *Synergistetes* were increased with increasing pollutant levels, while relative abundances of *Proteobacteria*, *Chloroflexi*, and *Thaumarchaeota* were decreased with increasing pollutant levels.

### 3.4. Effects of Environmental Variables on Bacterial Community

**Figure 5:**
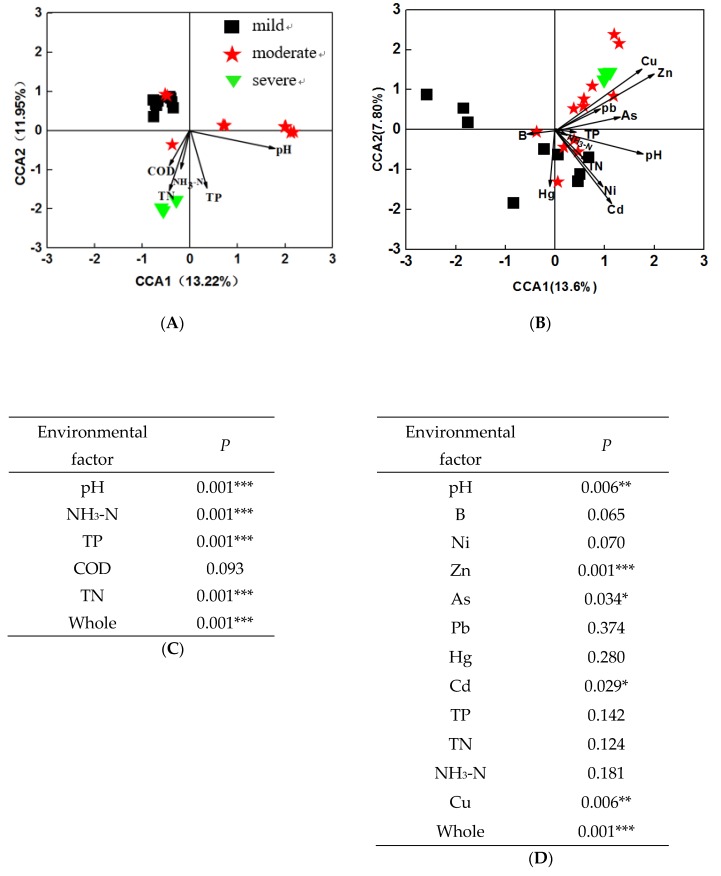
Canonical correspondence analysis (CCA) plot of bacterial communities and environmental parameters in water samples (**A**), and sediment samples (**B**). Model significance of water samples (**C**) and sediment samples (**D**).

To investigate the relationship between microbial community structure and environmental factors, CCA was performed across 24 water and sediment samples, respectively. The results showed that microbial communities were separated by the pollutant levels, and were significantly (*P* = 0.001) correlated with environmental factors, both in water and in sediment. In water samples (Figure 5A), the first axis explained 13.22% of the constrained variations in microbial communities, while the second axis explained 11.95%. Among these variables, four environmental variables, including pH, NH_3_-N, TP, and TN (*P* = 0.001) significantly correlated to bacterial communities (Figure 5C). Moreover, about 12% and 7.89% of the variations in sediment bacterial communities were explained by the first and second CCA axis, respectively. Among these variables, four environmental variables, including pH, Zn, Cu, As, and Cd were significantly correlated (*P* < 0.05) with bacterial communities (Figure 5D). These results indicated that environmental factors may significantly influence microbial structures in both water and sediment samples.

The linkage between predominant phyla and environmental factors were examined by mantel test. The results showed that water pH was significantly correlated with some phyla, such as Bacteroidetes, Firmicutes, Cyanobacteria / Chloroplast and Actinobacteria. TN, TP, NH_3_-N were significantly correlated with Proteobacteria (Table S3). In sediment samples Proteobacteria was correlated with Cu, Zn, NH_3_-N (Table S4). The results implied that nutrients selectively enriched bacteria from these phyla.

### 3.5. Ecological Processes in the Community Assembly

To further investigate the stochastic processes in shaping microbial community structure, stochastic ratios were calculated on the basis of taxonomic and phylogenetic metrics (Table 5). In water samples, the estimated NST was decreased with increasing pollutant levels (63.4%–21.4%), while stochastic ratios based on phylogenetic metrics appeared in a similar pattern to that of the estimated NST (39.1–16.3). The results indicated that the relative importance of stochastic processes in the governing community structure decreased substantially under increasing pollutant levels. These results indicated that environmental variations could act as a deterministic filtering factor to impose significant selection on the microorganisms so that the overall community-level stochasticity decreased along with the pollutant level. In sediment samples, the stochastic processes contributed to considerable portions of the community variations for the pollutant levels and affected the taxonomic (71.7%–15%) and phylogenetic (65.6%–10.3%) diversity. These results suggested that in sediment samples, stochastic processes could play more important roles in shaping a microbial community structure, which is consistent with the results from water samples described above.

## 4. Discussion

### 4.1. The Alpha Diversity Was Significantly Different for Different Pollution Levels of Ponds

Our initial hypothesis is that the diversity of microbial communities shifted under different pollutant levels. As we expected, our conclusion confirmed this hypothesis. Our results indicated that bacterial community diversity in water and sediment samples were significantly different among different levels polluted ponds. This revealed that the livestock wastewater discharge induced a great shift of diversity among bacterial groups, which agreed with a previous report suggesting that swine wastewater discharge evidently shifted bacterial diversity in the receiving river water [46]. In general, diversity is an important indicator of community stability, high diversity implied stability, and functional robustness in microbial communities [47,48]. Diversity of responses may also be a key determinant of ecosystem resilience in the face of anthropogenic pressure and environmental uncertainty [48]. In the water samples, mild ponds had the highest microbial diversity, which indicated that the bacterial community is more stable than other two groups. The severe ponds had higher microbial diversity than the moderate ponds, possibly because the wastewater discharged to ponds not long before we collected samples. In contrast to long-term polluted ponds, new wastewater emission may increase certain microbial communities, especially those functioning in livestock wastewater contaminant degradation, which causes an increasing of overall bacterial diversity [49,50]. However, the α diversity of bacterial communities in sediment samples decreased significantly with increasing wastewater contamination. This result is similar to that of Reference [51], which demonstrated that in heavily polluted fish farm sediments the diversity was reduced by a factor of 200 as compared to pristine sediments. These decreases in diversity imply that sediment microbial communities are not resistant or resilient to increasing pollution levels, thereby indicating that they do not converge to their previous composition [52].

### 4.2. Beta Diversity of Bacterial Were Significantly Changed under Different Pollution Levels of Ponds

We also hypothesized that the structure and composition of microbial communities shifted under different pollutant levels. As we expected, we have found significant differences in structure and composition of microbial communities under different pollutant levels. The results agreed well with a report of the Arga River showing that microbial community structures change definitively among different pollution levels from the discharge the sampling zone [53]. This clearly shows that pollutants can significantly affect the structure of microbial communities. In our study *Proteobacteria*, *Bacteroidetes*, and *Firmicutes* were the most dominant phyla in water samples. A very similar result was reported by Da et al. [54], who investigated the microbial community structure of swine wastewater. In addition, some other previous studies [55,56] also revealed that *Firmicutes* and *Proteobacteria* were the dominant phyla in the swine wastewater or pig intestinal microbiome. In sediment samples *Firmicutes*, *Proteobacteria*, *Chloroflexi*, and *Bacteroidetes* were detected to be the dominant phyla. This dominance pattern is similar to those found in lake and river estuarine sediments [10,11,37]. However, this pattern is distinct from those found in liquid swine manure during storage [57]. The results showed that water sediment systems share the characteristic profile of high bacterial amounts commonly observed in other aquatic ecosystems.

However, microbial population abundance exhibited differences across all the groups, which may be determined by a microbial community in the raw wastewater. Usually, environmental variations of freshwater, such as temperature, pH, salinity, and metal concentrations, would affect the taxonomical structure and relative abundance of species within bacterial communities in water body [10,13,14,58]. Hence, relative abundance of microbial community was influenced in large part by water quality.

### 4.3. Environmental Variations Play a Crucial Role in Community Assembly Processes

For our research, another hypothesis is that community assembly processes of water and sediment microbial communities have significant differences in different pollutant levels, which are affected by environmental variations. As we expected, based on the null model-based analyses results, we have observed that the stochastic ratio was decreased with the increasing pollution level, which indicates that deterministic processes played larger roles than stochastic ones in explaining microbial community taxonomic and phylogenetic compositions in severe polluted ponds. Such responses of these communities also show that community assembly is caused by environmental variations.

In addition, we found that the microbial communities in water and sediment are closely related to environmental variations. In water samples, pH, TP, TN, and NH_3_-N were considered as the crucial environmental factors driving the bacterial community shift. The concentrations of TP, TN, and NH_3_-N increased with the increasing pollution levels, which confirmed our previous inference. In addition, Zhang et al. [23] has also confirmed that pH, TP, and TN were selected as factors explaining the bacterial community variation in water samples in a heavily polluted urban river. In sediment samples pH, Zn, As, Cd, and Cu were selected as the crucial environmental factors. The mantel test results showed *Firmicutes* was significantly correlated with Cu and Zn (Table S4) in sediment samples. Chen et al. [59] indicated *Firmicutes* may be more sensitive to copper than commonly thought and may develop a community tolerance due to pollution, while the authors also showed the increased relative abundance in heavy metal-contaminated sediments indicates that they are more resistant to heavy metals in lake sediments. Thus, it is supposed that the abundance of *Proteobacteria* and *Actinobacteria* decreased with increasing pollution levels, but is less resistant or susceptible to heavy metals. For example, *Janthinobacterium* which is an important genus of *Betaproteobacteria* was found to be susceptible to heavy metals, such as Ag, Cu, Hg, and Pb [60]. So, the phyla which have an increased abundance with increasing pollution levels, such as *Bacteroidetes* (in water) *Firmicutes*, *Bacteroidetes*, *Cloacimonetes*, and *Synergistetes* (in sediment) may have adapted to the polluted environments and their diversity is maintained by various resistance mechanisms. The results indicated that the microbial community in the pollution ponds is sensitive to environmental factors and shaped by them. These analyses might provide some clues for us to unveil the molecular mechanism about how microbial communities shift to respond to the contamination of heavy metals.

Moreover, the influence of stochasticity in the mild polluted ponds is greater than in other groups. This result agrees with previous studies of microbial primary succession across diverse systems [61,62]. The mild polluted ponds had higher microbial diversity in both water and sediment samples compared to other two groups. These high levels of stochasticity and diversity may result from the initial physical characteristic of the water and sediment. The correlation analysis between environmental factors and α-diversities also demonstrated that the diversity index was significantly correlated with pH, TP, and NH_3_-N (water samples), as well as Cu, and Zn (sediment samples). Moreover, the environmental variations (e.g., pH, TP, and NH_3_-N concentrations) were not severe in the mild polluted ponds, so there has no strong environmental filter [61]. We considered that the environmental variations of the samples in mild polluted ponds led to weak selection and high immigration rates, so random ecological drift governs spatio-temporal variations in abundances of bacterial communities. However, the ecological stochasticity and determinism estimated using the framework with the null model should be viewed as statistically proximate rather than ultimate forces in shaping community diversity and structure. Thus, the model needs sufficient biological replication (e.g., >6) to ensure sufficient statistical power [43]. Meanwhile, further research is needed to examine whether the declining importance of stochastic processes identified in this study are applicable to other studies accompanied by large amounts of data or other ecosystems. 

In general, we found there has significant differences of community assembly processes at different pollution levels, while environmental variations may simultaneously be crucial factors affecting the bacterial community assembly process. We speculate that environmental factors affect the enrichment and inhibition of microbial communities, which leads to an assembly process for the communities altered.

## 5. Conclusions

In summary, we found that pollution levels significantly altered the microbial diversity, composition, and structure. In addition, since the environmental variations act as a deterministic filtering factor to impose significant selection on microorganisms, the overall community-level stochasticity decreased with increasing pollution levels, indicating the communities could converge more quickly to a community state with less stochasticity under increasing pollution levels. Any ecosystem restoration programs must consider approaches to facilitate both stochastic and deterministic processes. So, these findings could enhance our understanding of bacterial community assembly and underlying ecological processes in rural ponds. That may be useful for the choice of suitable bioremediation technologies for rural ponds influenced by different pollutant levels of livestock wastewater.

## Figures and Tables

**Table 1:** Information about the sampling ponds.

	Total Area (m^2^)	Water Area (m^2^)	Water Depth (m)	Water Capacity (m^3^)	Sediment Depth (m)	Sediment Volume (m^3^)
1#	11,291.9	4848.3	1.74	8436.1	1.8	8727
2#	6517	4784.3	1.272	6085.7	2.4	11,482.4
3#	17,021.2	9213.2	1.08	9950.3	1.6	14,741.2
4#	898.4	409.7	1.2	491.7	1.8	157.7
5#	1197.6	857.1	2.64	2262.8	2.4	2057.4
6#	3562.9	2358.2	0.95	2240.3	1.2	2829.9
7#	1628.8	1044.4	1.8	1880	1.56	1629.3
8#	2547.7	1539.4	6.28	9667.5	2.4	3694.6

**Table 2:** Environmental variables of water samples with different pollutant levels.

	Mild	Moderate	Severe	Standard (mg/L)^a^
pH	7.10 ± 0.06A	8.16 ± 0.18B	7.69 ± 0.18AB	6~9
NH_3_-N	3.39 ± 0.81A	41.79 ± 8.09B	451.33 ± 27.53C	2.0
TP	9.64 ± 2.52A	38.63 ± 7.47B	72.23 ± 7.61C	0.4
COD	634.78 ± 264.52A	422.25 ± 72.57A	1186.67 ± 455.81A	40
TN	76.04 ± 34.00A	97.11 ± 13.06A	687.67 ± 142.54B	2.0
comprehensive pollution index range	11.63–24.87	33.00–37.95	156.12	

^a^ environmental quality standards for surface water (GB3838-2002). Environmental variables were presented as mean ± standard error (SE). A and B indicate significant changes among three groups of ponds. The significance of difference was analyzed by least significant difference (LSD).

**Table 3:** Physical and chemical properties in sediment samples.

Unit (mg/L)	Mild	Moderate	Severe
B (—)^b^	35.84 ± 3.91A	29.32 ± 2.06A	34.87 ± 1.57A
Cr (500)	27.35 ± 1.89AB	21.00 ± 1.37A	30.80 ± 0.60B
Ni (100)	22.31 ± 1.70AB	18.66 ± 1.25A	25.93 ± 0.91B
Cu (500)	29.47 ± 3.90A	96.75 ± 25.14A	70.82 ± 6.97A
Zn (1200)	86.79 ± 7.49A	204.81 ± 25.96B	213.70 ± 16.63B
As (30)	7.04 ± 0.65A	6.65 ± 1.02A	9.39 ± 1.01A
Cd (3)	0.37 ± 0.06A	0.31 ± 0.02A	0.24 ± 0.02A
Pb (300)	19.62 ± 1.02A	27.91 ± 7.98A	33.77 ± 12.22A
Hg (3)	2.74 ± 1.35A	0.83 ± 0.16A	0.82 ± 0.1A
TP	812.44 ± 25.63A	956.67 ± 69.7A	751.33 ± 72.84A
TN	1876.22 ± 73AB	2163.83 ± 192.08B	1415.67 ± 85.93A
NH_3_-N	93.52 ± 4.14A	100.30 ± 7.27A	80.59 ± 2.81A
pH (5.5–8.5)	7.93 ± 0.13A	8.24 ± 0.1A	8.01 ± 0.33A
OM (≥20)	7522.67 ± 599.74AB	13950.25 ± 2084.48B	6273.00 ± 403.35A

OM: Organic Matter. b: grade A of the Control Standards of Pollutants in sediment for Agricultural use (GB4284-2018). A and B indicate significant changes among three groups of ponds. The significance of difference was analyzed by using least significant difference (LSD).

**Table 4:** Dissimilarity tests of water and sediment microbial community based on Bray Curtis and Jaccard distances.

Groups	Bray Curtis				Jaccard			
	MRPP		PERMANOVA		MRPP		PERMANOVA	
water samples	δ	*P*	F	*P*	δ	*P*	F	*P*
mild vs. moderate	0.7651	0.003**	2.9581	0.005**	0.8013	0.001***	3.2270	0.001***
mild vs. severe	0.6388	0.005**	4.3320	0.006**	0.7479	0.003**	2.6988	0.004**
moderate vs. severe	0.7176	0.011*	3.6158	0.005**	0.7625	0.001***	3.0440	0.005**
sediment samples								
mild vs. moderate	0.6633	0.001***	2.9240	0.007**	0.7718	0.001***	2.6000	0.001***
mild vs. severe	0.5908	0.005**	4.2474	0.012**	0.7302	0.003**	3.2429	0.008**
moderate vs. severe	0.6077	0.025*	1.6452	0.092	0.7652	0.015*	1.6279	0.035*

Significant correlation coefficient at: * *P* ≤ 0.05; ** *P* ≤ 0.01; *** *P* ≤ 0.001.

**Table 5:** Normalized stochasticity ratio (NST) based on Jaccard distance about the bacterial community structure and significance test of the similarity between the microbial communities and null model simulations in three groups of ponds.

Groups	NST (Null Model Based on Taxonomic)		Null Model Based on Phylogenetic				
	Group	Stochasticity Process (%)	Mean of Observed Similarity	Mean of Null Expected Similarity	Stochasticity Process (%)	F	*P* ^c^
Water	mild	0.634	0.147	0.057	0.391	25.567	<0.001
	moderate	0.403	0.125	0.032	0.257	2.846	0.106
	severe	0.214	0.352	0.057	0.163	0.469	0.531
Sediment	mild	0.717	0.148	0.097	0.656	79.074	<0.001
	moderate	0.540	0.172	0.060	0.349	4.613	0.043
	severe	0.150	0.371	0.038	0.103	4.617	0.098

^c^ a Permutational multivariate analysis of variance (PERMANOVA) was conducted.

## Supplementary Materials

The following are available online at https://www.mdpi.com/2076-2607/8/2/311/s1, Table S1. Spearman’s ranked correlation between the alpha diversity and water quality indexes. Table S2. Spearman’s ranked correlation between the alpha diversity and environmental variables in sediment samples. Table S3. Mantel test of dominant bacteria at phylum level among different pollutant levels in water samples. Table S4. Mantel test of dominant phylum and environmental factors in sediment.
