# Supplementary material for: Pollution Gradients Altered the Bacterial Community Composition and Stochastic Process of Rural Polluted Ponds
Source: Microorganisms. 2020 Feb 24;8(2):311. doi: 10.3390/microorganisms8020311 (PMC7074964; doi:10.3390/microorganisms8020311)
Supplement: Supplementary file 1 [file microorganisms-08-00311-s001.pdf]

microorganisms

Supplementary Materials

Table S1. Spearman's ranked correlation between the alpha diversity and water quality indexes.

	Shannon	Observed richness	Chao 1
pH	0.016*	<0.001***	<0.001***
NH_3-N	0.314	0.040*	0.021*
TP	0.334	0.022*	0.014*
COD	0.217	0.866	0.831
TN	0.479	0.812	0.589

Significant correlation coefficient at: *$P \leq 0.05$; **$P \leq 0.01$; ***$P \leq 0.001$.

Table S2. Spearman's ranked correlation between the alpha diversity and environmental variables in sediment samples.

	Shannon	Inv_Simpson	Observed richness	Chao 1
pH	0.348	0.467	0.243	0.378
B	0.694	0.908	0.534	0.306
Cr	0.821	0.691	0.71	0.869
Ni	0.659	0.575	0.595	0.691
Cu	0.003***	0.025*	0.001***	0.001**
Zn	0.003**	0.018*	0.003**	0.003**
As	0.17	0.15	0.171	0.285
Cd	0.716	0.339	0.891	0.981
Pb	0.576	0.64	0.609	0.343
Hg	0.558	0.494	0.434	0.831
TP	0.977	0.801	0.856	0.915
TN	0.609	0.354	0.78	0.703
NH_3-N	0.777	0.461	0.86	0.84
OM	0.65	0.956	0.539	0.606

Significant correlation coefficient at: *$P \leq 0.05$; **$P \leq 0.01$; ***$P \leq 0.001$.

Table S3. Mantel test of dominant bacteria at phylum level among different pollutant levels in water samples.

Environmental factor	Proteobacteria	Bacteroidetes	Firmicutes	Cyanobacteria/Chloroplast	Actinobacteria
	p	*p*	*p*	*p*	*p*
pH	0.228	0.001***	0.001***	0.001***	0.003**
NH_3-N	0.007**	0.266	0.040*	0.047*	0.009**
TP	0.009**	0.068	0.031*	0.039*	0.245
COD	0.290	0.495	0.097	0.112	0.224
TN	0.004**	0.256	0.128	0.123	0.005**

Table S4. Mantel test of dominant phylum and environmental factors in sediment.

Environmental factor	Proteobacteria	Bacteroidetes	Firmicutes	Chloroflexi	Actinobacteria	Acidobacteria
	p	*p*	*p*	*p*	*p*	*p*
pH	0.440	0.109	0.234	0.923	0.178	0.019*
B	0.698	0.133	0.315	0.859	0.752	0.322
Cr	0.103	0.290	0.236	0.264	0.041*	0.297
Ni	0.204	0.294	0.272	0.267	0.064	0.274
Cu	0.024*	0.165	0.036*	0.210	0.014*	0.169
Zn	0.004**	0.098	0.010**	0.531	0.020*	0.100
As	0.131	0.169	0.118	0.029*	0.031*	0.033*
Cd	0.963	0.130	0.241	0.183	0.842	0.262
Pb	0.243	0.715	0.504	0.594	0.294	0.544
Hg	0.850	0.140	0.074	0.437	0.630	0.307
TP	0.131	0.768	0.616	0.150	0.052	0.610
TN	0.103	0.358	0.420	0.185	0.018*	0.317
NH_3-N	0.036*	0.326	0.236	0.038*	0.011*	0.272
OM	0.105	0.302	0.209	0.200	0.007**	0.538

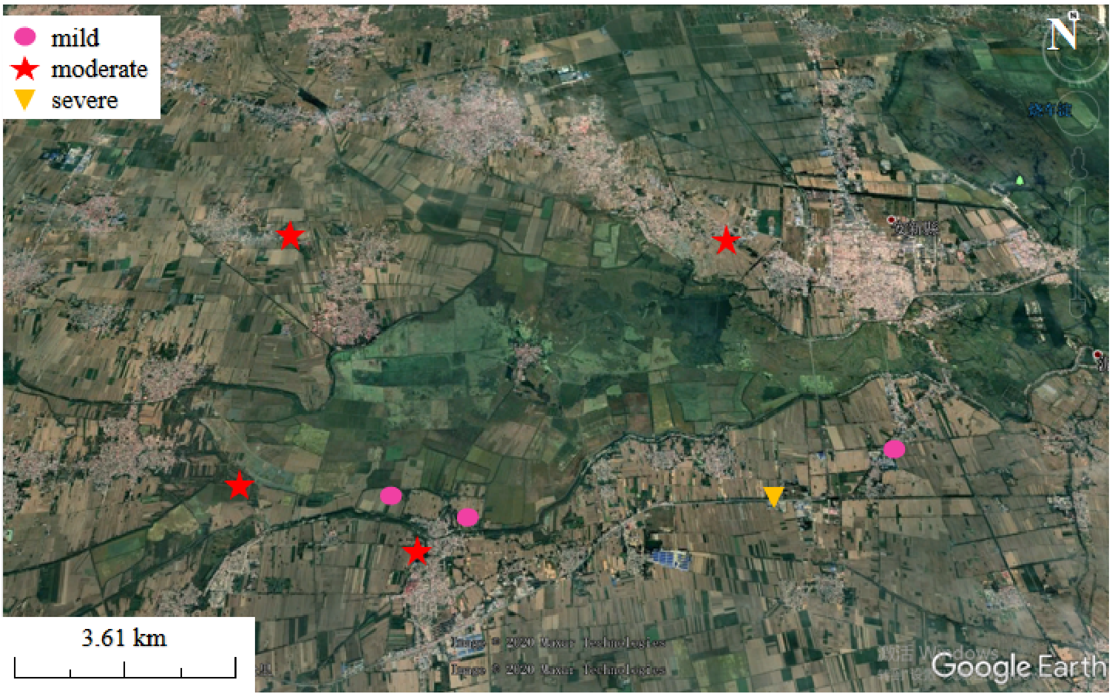

Figure S1. The geological locations of sampling sites.
